# EUPATI Guidance for Patient Involvement in Medicines Research and Development: Health Technology Assessment

**DOI:** 10.3389/fmed.2018.00231

**Published:** 2018-09-06

**Authors:** Amy Hunter, Karen Facey, Victoria Thomas, David Haerry, Kay Warner, Ingrid Klingmann, Matthew May, Wolf See

**Affiliations:** ^1^European Patients' Academy on Therapeutic Innovation (EUPATI); ^2^Genetic Alliance UK, London, United Kingdom; ^3^University of Edinburgh, Edinburgh, United Kingdom; ^4^The National Institute for Health and Care Excellence, London, United Kingdom; ^5^European Aids Treatment Group, Brussels, Belgium; ^6^GSK, London, United Kingdom; ^7^European Forum for Good Clinical Practice, Brussels, Belgium; ^8^European Patients Forum, Brussels, Belgium; ^9^Bayer, Berlin, Germany

**Keywords:** patient involvement, patient engagement, participation, health technology assessment, HTA, EUPATI

## Abstract

The main aim of health technology assessment (HTA) is to inform decision making by health care policy makers. It is a systematic process that evaluates the use of health technologies and generally involves a critical review of international evidence related to clinical effectiveness of the health technology vs. the best standard of care. It can also include an evaluation of cost effectiveness, and social and ethical impacts in the local health care system. The HTA process advises whether or not a health technology should be used, and if so, how it is best used and which patients are most likely to benefit from it. The importance of patient involvement in HTA is becoming widely recognized, for scientific and democratic reasons. The extent of patient involvement in HTA varies considerably across Europe. Commonly HTA is still focused on quantitative evidence to determine clinical and/or cost effectiveness, but the interest in understanding patients' experiences and preferences is increasing. Some HTA bodies provide support for participation in their processes, but again this varies widely across Europe. The involvement of patients in HTA is determined at the national and regional level, and is not subject to any European-wide legislation. The guidance text presented in this article was developed as part of the work of the European Patients' Academy on Therapeutic Innovation (EUPATI) and covers the interaction between HTA bodies and patients and their representatives when medicines are being assessed. Other EUPATI guidance documents relate to patient involvement in pharmaceutical industry-led research and development, ethics committees, and regulatory authorities. The guidance provides recommendations for activities to support patient involvement in HTA bodies and specific guidance for individual HTA processes. It seeks to improve patient involvement, using the outcomes of published research and consensus-building exercises. It also draws on good practice examples from individual HTA bodies. The guidance is not intended to be prescriptive and should be used according to specific circumstances, national legislation, or the unique needs of each interaction. This article represents the formal publication of the HTA guidance text with discussion about recent progress in, and continuing barriers to, patient involvement in HTA.

## Introduction

There is considerable enthusiasm for drawing on patients' knowledge and experience across the cycle of research and development of medicines in order to benefit patients themselves and the companies and authorities operating in medicines development. Patients bring to the table their unique lived experience of specific conditions and of their care and medication, and are motivated to engage. The European Patients' Academy on Therapeutic Innovation (EUPATI)—a partnership of patient organizations, universities, not-for-profit organizations, and pharmaceutical companies—offers education and training to improve the skills and capacity for patients to become involved in a meaningful way with every stage of medicines development. EUPATI recognized a lack of Europe-wide guidance for stakeholders wishing to support patient involvement, and has addressed this gap by developing a set of four guidance documents covering industry-led research and development, ethics committees, regulatory authorities, and health technology assessment (HTA)^1^.

This paper represents the official publication of the guidance document on patient involvement in HTA (1), along with a discussion about progress and new resources since the guidance was developed, and about the continuing barriers to meaningful patient involvement. The guidance text itself forms the main body of this paper. The text includes a set of introductory “overarching principles” applicable throughout the medicines research and development process; the guidance disclaimer; the scope of the guidance; an explanation of the definition of the term “patient” adopted by EUPATI; the rationale for developing the guidance; background information about patient involvement in HTA in Europe; and the ultimate objectives of the guidance. These sections are followed by the recommendations (suggested working practices and patient involvement activities).

There is a substantial literature on the scientific and democratic reasoning for meaningful patient involvement in HTA, and on the greatly varying levels of support provided by HTA bodies across Europe and worldwide to optimize patient involvement (2–5).

The guidance document draws on the outcomes of published research and consultations, and on good practice examples from HTA agencies. It recommends activities to support patient involvement in HTA bodies and specific guidance for individual HTA processes. The bedrock for the guidance is the set of values identified by the HTAi (an international society for the promotion of health technology assessment) in its international consensus-building exercise (6). Patient organizations, academia, HTA agencies, and industry contributed to the exercise, which received input from 150 respondents in 39 countries. The values, defined in full in the text of the guidance document, are: Relevance; Fairness; Equity; Legitimacy; Capacity building.

The guidance document is fully referenced in its online form but references have been removed from the guidance text embedded here to avoid confusion with citations specific to this article. The full online guidance text also includes a list of freely available resources.

## The EUPATI guidance on patient involvement in HTA

### Overarching principles for patient involvement throughout the medicines research and development process

The European Patients' Academy (EUPATI) is a pan-European Innovative Medicines Initiative (IMI) project of 33 organizations with partners from patient organizations, universities, not-for-profit organizations, and pharmaceutical companies. Throughout EUPATI the term “patient” references all age groups across conditions. EUPATI does not focus on disease-specific issues or therapies, but on the process of medicines development in general. Indication-specific information, age-specific or specific medicine interventions are beyond the scope of EUPATI and are the remit of health professionals as well as patient organizations. To find out more visit www.eupati.eu/.

The great majority of experts involved in the development and evaluation of medicines are scientists working both in the private and public sector. There is an increasing need to draw on patient knowledge and experience in order to understand what it is like to live with a specific condition, how care is administered and the day-to-day use of medicines. This input helps to improve discovery, development, and evaluation of new effective medicines.

Structured interaction with patients of all age groups and across conditions, their representatives and other stakeholders is necessary and allows the exchange of information and constructive dialog at national and European level where the views from users of medicines can and should be considered. It is important to take into account that healthcare systems as well as practices and legislation might differ.

We recommend close cooperation and partnership between the various stakeholders including healthcare professionals' organizations, contract research organizations, patients' and consumers' organizations, academia, scientific and academic societies, regulatory authorities and HTA bodies and the pharmaceutical industry. Experience to date demonstrates that the involvement of patients has resulted in increased transparency, trust and mutual respect between them and other stakeholders. It is acknowledged that the patients' contribution to the discovery, development and evaluation of medicines enriches the quality of the evidence and opinion available (1).

Existing codes of practice for patient involvement with various stakeholders do not comprehensively cover the full scope of research and development (R&D). The EUPATI guidance documents aim to support the integration of patient involvement across the entire process of medicines research and development.

These guidance documents are not intended to be prescriptive and will not give detailed step-by-step advice.

EUPATI has developed these guidance documents for all stakeholders aiming to interact with patients on medicines research and development (R&D). Users may deviate from this guidance according to specific circumstances, national legislation or the unique needs of each interaction. This guidance should be adapted for individual requirements using best professional judgment.

There are four separate guidance documents covering patient involvement in:

- Pharmaceutical industry-led medicines R&D
- Ethics committees
- Regulatory authorities
- Health technology assessment (HTA).

Each guidance suggests areas where at present there are opportunities for patient involvement. This guidance should be periodically reviewed and revised to reflect evolution.

### This guidance covers patient involvement in health technology assessment (HTA)

All subsequently developed guidance should be aligned with existing national legislation covering interactions as stated in the four EUPATI guidance documents.

### Disclaimer

EUPATI has developed this guidance for all stakeholders aiming to interact with patients in HTA.

This guidance document is not intended to be prescriptive and will not give detailed step-by-step advice. This guidance should be used according to specific circumstances, national legislation or the unique needs of each interaction. This guidance should be adapted for individual requirements using best professional judgment.

Where this guidance offers advice on legal issues, it is not offered as a definitive legal interpretation and is not a substitute for formal legal advice. If formal advice is required, involved stakeholders should consult their respective legal department if available, or seek legal advice from competent sources.

EUPATI will in no event be responsible for any outcomes of any nature resulting from the use of this guidance.

The EUPATI project received support from the Innovative Medicines Initiative Joint Undertaking under grant agreement n° 115334, resources of which are composed of financial contribution from the European Union's Seventh Framework Programme (FP7/2007-2013) and EFPIA companies.

### Scope of the eupati guidance on patient involvement in HTA

**Figure 1:**
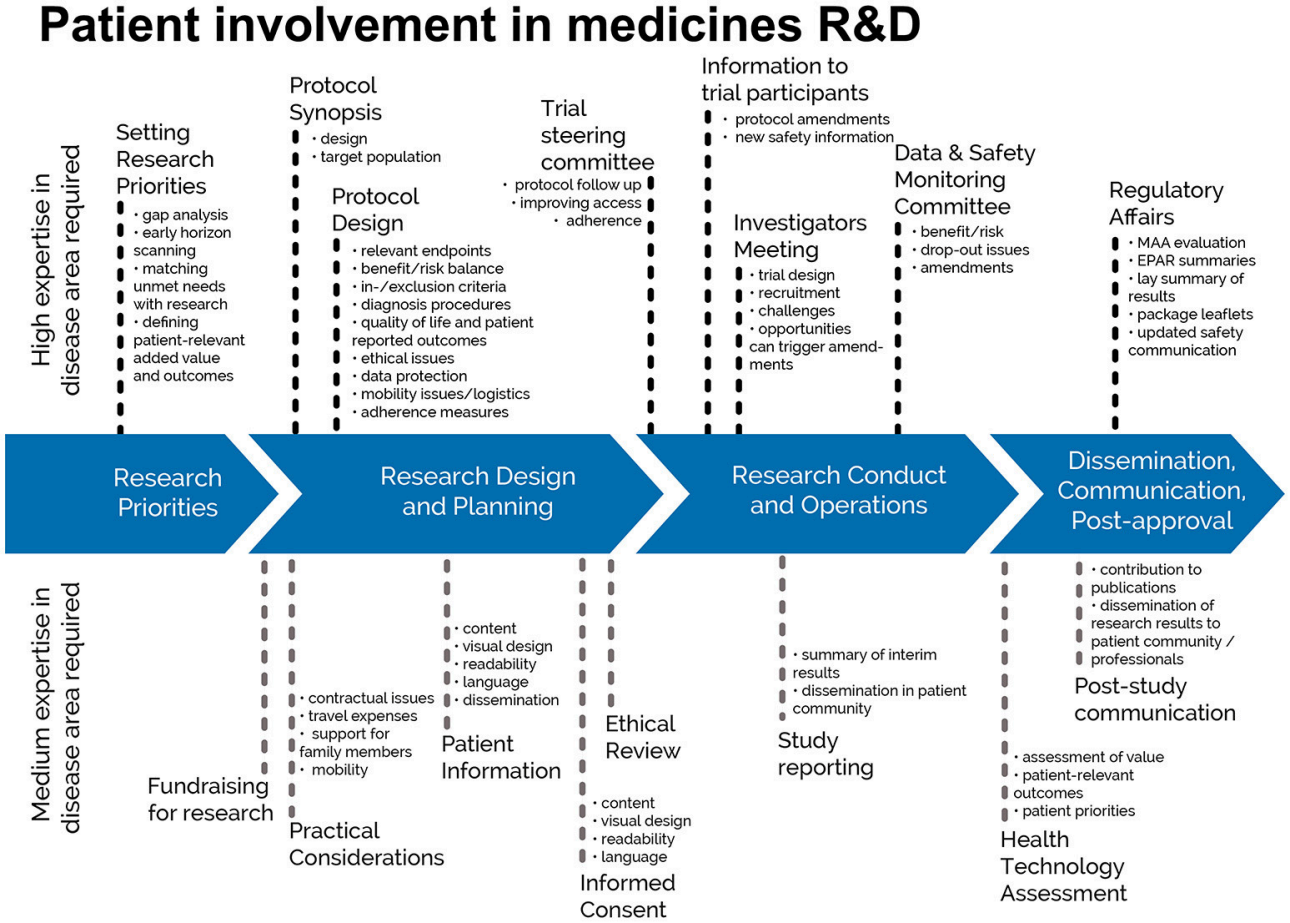
Patient involvement in medicines R&D. Patients can be involved across the process of medicines R&D. This diagram created by Geissler, Ryll, Leto, and Uhlenhopp identifies some existing areas in which patients are involved in the process. It distinguishes between the level of expertise in a disease area that is required and the different areas where involvement can take place. Copyright: EUPATI, under a Creative Commons Attribution-NonCommercial-ShareAlike 4.0 International (CC BY-NC-SA 4.0) licence. Used with permission.

This European guidance covers the interaction between HTA bodies and patients^2^ in relation to medicines for human use. HTA processes are applied to interventions other than medicines, but they are not the focus of this guidance, in line with the remit of EUPATI. Figure 1 indicates where patients can be involved currently throughout the medicines R&D lifecycle; however, this is not meant to limit involvement, and opportunities may change and increase over time.

The guidance focuses on participation in the HTA process, and excludes the scientific collection of patient perspectives (i.e., it excludes quantitative and qualitative research on the perspectives, experiences and preferences of patients).

### Defining “patient”

The term “patient” is often used as a general, imprecise term that does not reflect the different types of input and experience required from patients, patient advocates, and patient organizations in different collaborative processes.

In order to clarify terminology for potential roles of patient interaction presented in this and the other EUPATI guidance documents, we use the term “patient” which covers the following definitions:

- “Individual Patients” are persons with personal experience of living with a disease. They may or may not have technical knowledge in R&D or regulatory processes, but their main role is to contribute with their subjective disease and treatment experience.
- “Carers” are persons supporting individual patients such as family members as well as paid or volunteer helpers^3^.
- “Patient Advocates” are persons who have the insight and experience in supporting a larger population of patients living with a specific disease. They may or may not be affiliated with an organization.
- “Patient Organization Representatives” are persons who are mandated to represent and express the collective views of a patient organization on a specific issue or disease area.
- “Patient Experts,” in addition to disease-specific expertise, have the technical knowledge in R&D and/or regulatory affairs through training or experience, for example EUPATI Fellows who have been trained by EUPATI on the full spectrum of medicines R&D.

There may be reservations about involving individual patients in collaborative activities with stakeholders on grounds that their input will be subjective and open to criticism. However, EUPATI, in line with regulatory authorities, instills the value of equity by not excluding the involvement of individuals. It should be left to the discretion of the organization(s) initiating the interaction to choose the most adequate patient representation in terms of which type of patient for which activity. Where an individual patient will be engaged it is suggested that the relevant patient organization, where one exists, be informed and/or consulted to provide support and/or advice.

The type of input and mandate of the involved person should be agreed in any collaborative process prior to engagement.

### Rationale for the guidance

HTA stands for Health Technology Assessment. The main aim of HTA is to inform decision making by health care policy makers. It is a systematic process that considers health technologies (such as medicines or medical devices) and can involve a review of:

- Clinical effectiveness (how well a medicine will work in the local health system compared to the best standard of care)
- Cost effectiveness (the long term costs and benefits of a medicine compared to the best standard of care)
- Social and ethical impacts on the health care system and the lives of individual patients.

The process advises whether or not a health technology should be used, and if so, how it is best used and which patients are most likely to benefit from it. Assessments vary, but most look at the health benefits and risks of using the technology. They can also look at costs and any other wider impacts that the technology may have on a population or on a society.

HTA assesses international evidence but applies it to the local health care setting to understand the added value of a new medicine in that health care system. HTAs are performed at national, regional or hospital level.

The importance of patient involvement in HTA is becoming widely recognized. Patients are directly affected by HTA decisions—they are key stakeholders, and have a “democratic right” to be involved. HTA can be considered to be a bridge between scientific evidence and decision-making and as a result there are both scientific and democratic reasons that support effective patient involvement in HTA.

Patients can provide information and insight, about the impact of their condition and treatments on their daily lives that is not available elsewhere. Patients are in a unique position to describe the outcomes that matter to them, to challenge presumptions about their health aspirations and to inform HTA processes about the potential positive or negative effects of new and existing technologies—on their health and on their ability to live and work.

### Background

The extent of patient involvement in HTA varies considerably between countries and regions in Europe. Commonly HTA is still focused on quantitative evidence to determine clinical and/or cost effectiveness, although there are instances of active patient support.

The extent and nature of support for patients provided by HTA bodies, to optimize patient involvement in their processes, also varies a great deal.

The involvement of patients in HTA is determined at the national and regional level, and is not subject to any European legislation.

HTA bodies and patient organizations have reported a positive impact of patient involvement on the processes and/or outcomes of HTA. Although systematic research into the impact of different approaches of patient involvement is scarce those case studies that are available make the impact of patient involvement explicit. Bodies such as HTAi and ISPOR are working to develop the evidence base and provide repositories of materials for patient involvement.

The HTA Core Model® (version 3.0) produced by EUnetHTA (a network of government appointed organizations, regional agencies and non-for-profit organizations that produce or contribute to HTA in Europe) provides a detailed technical guideline for HTA agencies, outlining the types and sources of evidence required for HTA. Patients are included as potential sources of evidence. The HTA Core Model® is aimed at professionals with HTA expertise and the topic of patient involvement in HTA processes more widely is outside its scope.

There is therefore a need for a Europe-wide guidance on patient interaction in HTA to promote good practice and to complement the work of EUnetHTA.

### Objectives of the EUPATI guidance on patient involvement with HTA

The following values are recognized in the guidance, and worked toward through the adoption of the suggested working practices. The values, given in the table below, are one output of a consensus-building exercise by HTAi. Patient organizations, academia, HTA agencies and industry contributed to the exercise, which received input from 150 respondents in 39 countries.

The values are:

**Table:** 


Relevance	Patients have knowledge, perspectives and experiences that are unique and contribute to essential evidence for HTA.
Fairness	Patients have the same rights to contribute to the HTA process as other stakeholders and have access to processes that enable effective engagement.
Equity	Patient involvement in HTA contributes to equity by seeking to understand the diverse needs of patients with particular health issues, balanced against the requirements of a health system that seeks to distribute resources fairly among all users.
Legitimacy	Patient involvement facilitates those affected by the HTA recommendations/decision to participate in HTA; contributing to the transparency, accountability and credibility of the decision-making process.
Capacity building	Patient involvement processes address barriers to involving patients in HTA and build capacity for patients and HTA organizations to work together.

### Recommendations

#### Suggested working practices

The working methods recommended for HTA agencies and patient organizations in this section arise from several sources. The primary sources are the set of quality standards from the HTAi consensus-building exercise, reviews of individual HTA agencies and the European Patients' Forum (EPF) survey of patient involvement in HTA in Europe. Specific patient involvement activities that are employed or planned by HTA agencies are given in section “Suggested patient involvement activities.”

For HTA bodies that are new to patient involvement, a step-wise approach to introducing new working methods and activities may be the most successful. Prioritization of specific working methods and activities should be decided on by individual HTA bodies with patients and other stakeholders.

In order to achieve the objectives identified above, the following should be considered by HTA bodies:

- Should have a strategy that outlines the processes and responsibilities for those working in HTA and serving on HTA committees, to effectively involve patients.
- Should designate appropriate resources to ensure and support effective patient involvement in HTA.
- HTA participants (including researchers, staff, HTA reviewers and committee members) should receive training about appropriate involvement of patients and consideration of patients' perspectives throughout the HTA process.
- Patients should be given the opportunity to receive mentoring and training so that they can contribute most effectively to HTA.
- Patient involvement processes in HTA should be regularly reflected on and reviewed, taking account of the experiences of all those involved, with the intent to continuously improve the processes.
- Should work to align internal and external stakeholders on the objectives of patient input processes.
- Should have proactive communications strategies to effectively reach, inform and enable a wide range of patients to participate fully in each HTA, including making public the criteria and processes they use to reach decisions.
- Should have clear timelines established for each HTA with advance notice of deadlines to ensure that appropriate input from a wide range of patients can be obtained.
- For each HTA, should identify a staff member whose role is to support patients to contribute effectively to HTA.
- In each HTA, patients' perspectives and experiences should be documented and the influence of patient contributions on conclusions and decisions should be reported.
- Should provide feedback to patients who have contributed to an HTA, to share what contributions were most helpful and provide suggestions to assist their future involvement.
- Each HTA should use accessible language in documents and other materials for the patients involved.
- Should give patients the opportunity to participate other than through making submissions to specific HTAs.
- Should develop frameworks to systematically incorporate patient input to HTAs.
- Should make systems for written submissions easy to use and appropriate support should be offered to individuals making submissions.

In order to achieve the objectives identified above, the following should be considered by patient organizations:

- Ensure those speaking on your behalf are trained in HTA, to have knowledge of both its role in healthcare resource allocation and scientific and cost-effectiveness aspects.
- Where there are no or few patient involvement activities, approach HTA agencies pro-actively and suggest how patient involvement can be achieved through clear proposals.
- Understand the HTA processes: meet with HTA staff, follow guidelines and deadlines, use glossaries if available.
- Learn from the experience of other patient organizations and collaborate with them.
- Remain transparent: declare (publish) and diversify your financial support, and have a clear and explicit framework for cooperating with industry.

#### Suggested patient involvement activities

The suggested activities outlined in this section are examples of specific mechanisms to involve patients. All are already practized (or planned) by one or more HTA bodies. They are drawn from publications from HTAi, EPF, the International Network for Agencies for HTA (INAHTA), individual HTA agencies and academic reviews.

The following text uses the term “patient” to refer to the different categories defined above.

#### General HTA process

**Figure 2:**
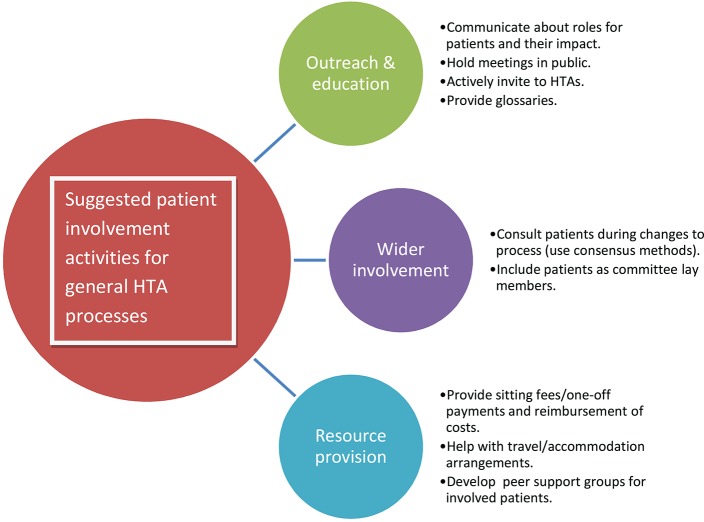
Suggested patient involvement activities for general HTA processes. The activities listed here will help implement the recommended working methods for the HTA process in general. The list does not aim to be exhaustive but to provide initial ideas. Copyright: EUPATI, under a Creative Commons Attribution-NonCommercial-ShareAlike 4.0 International (CC BY-NC-SA 4.0) licence. Used with permission.

Aimed at HTA organizations, the activities listed here, and summarized in Figure 2, will help implement the recommended working methods for the HTA process in general. The list does not aim to be exhaustive but to provide initial ideas.

##### Outreach and education

- Produce guidance materials on the different roles patients may take within HTA processes.
- Provide a single point of contact for patient involvement issues.
- Give presentations and training workshops for patient organization representatives, about HTA and patient involvement.
- Evaluate and communicate about the impact patients have had, to demonstrate that they can make a difference.
- Hold HTA meetings in public as far as possible.
- Provide a glossary in relevant language(s) of HTA-specific terms.
- Advertise forthcoming HTAs including alerting through regular bulletins, and actively invite patient organizations to take part.
- Support the development of peer support groups for patients involved with individual HTA bodies.

##### Wider involvement

- Include patients when consulting on potentially significant changes to HTA processes.
- Consider the use of participatory approaches, such as Citizen's Jury or consensus conference methods, during development of HTA processes.
- Include patient experts as lay members, or in addition to lay members, of HTA committees not just as contributors to specific HTAs. Give these members full voting rights.

#### For individual HTAs

**Figure 3:**
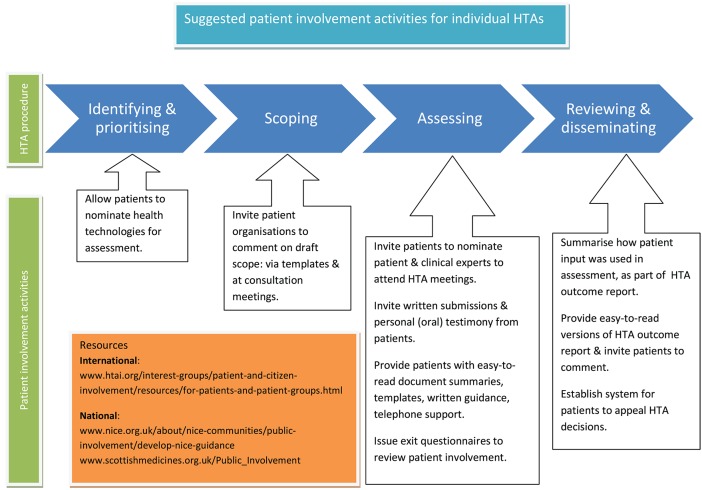
Suggested patient involvement activities for individual HTAs. The activities listed here are aimed at HTA organizations, to help implement the recommended working methods for individual HTAs. The list does not aim to be exhaustive but to provide initial ideas. Copyright: EUPATI, under a Creative Commons Attribution-NonCommercial-ShareAlike 4.0 International (CC BY-NC-SA 4.0) licence. Used with permission.

The activities listed here, and summarized in Figure 3, are again aimed at HTA organizations, to help implement the recommended working methods for individual HTAs. The list does not aim to be exhaustive but to provide initial ideas.

##### Identifying and prioritizing which technologies to assess

- Develop a system for patients to nominate technologies for HTA.

##### Scoping (developing a framework for an individual HTA)

- Consult with patient organizations on the draft scope using templates for written submissions.
- Invite patient organizations to oral consultation meetings to take part in discussion on the HTA scope.

##### Assessing and developing recommendations/guidelines

- Invite patient organizations to nominate patient and clinical experts to attend HTA committee meetings.
- Invite written submissions from individual patients/carers and patient organizations to form part of the evidence base considered by the committee.
- Provide templates, guidance documents, and telephone support for those completing written submissions, and preparing to act as patient experts at meetings.
- Invite oral submissions from individual patients/carers at committee meetings i.e., personal testimony.
- Provide easy to read summaries of documentation sent out ahead of individual HTAs.
- Give free access for patients to any original publications that will form part of the HTA evidence.
- Develop an exit questionnaire for patients attending meetings, to be issued after each HTA, and feed results into the overall review of patient involvement.

##### Reviewing and disseminating HTA outcomes

- Summarize patient input in HTA outcome documents, and how it was used in reaching the final recommendation. When suggestions from patients were not included in the final recommendation, provide a properly justified written explanation.
- Provide lay language versions of HTA outcome documents.
- Invite written comment on drafts of HTA outcomes from patients taking part in the HTA, and from others who were unable to take part (for example for health reasons).
- Develop and disseminate a clear system for patients to appeal HTA decisions.
- Involve patients in the review of patient involvement processes.

### Compensation

It should be recognized that in many situations patients involved in activities do so voluntarily either as an individual but also when a member of an organization. Consideration should therefore be given to:

- Compensate for their total time invested plus expenses.
  - Any compensation offered should be fair and appropriate for the type of engagement. Ideally travel costs would be paid directly by the organizing partner, rather than being reimbursed.
- Covering the costs incurred by patient organizations when identifying or supporting patients for involvement in activities (i.e., peer support groups, training and preparation) should also be considered.
- Help organize the logistics of patient participation, including travel and/or accommodation.

Compensation also includes indirect benefits in kind (such as the a patient organization providing services free of charge) or any other non-financial benefits in kind provided to the patient/patient organization (such as training sessions, agency services, the setting up of web sites).

All parties should be transparent about any compensation arrangements.

### Written agreement

At a minimum a written agreement should clearly define: a description of the activity and its objectives, the nature of the interaction during the activity, consent (if relevant), release, confidentiality, compensation, data privacy, compliance, declaration of conflict of interest, timelines. Interaction may only proceed on the basis of a written agreement that at a minimum spells out the basic elements of the collaboration (e.g., rules of engagement, compliance, intellectual property, financial payments).

Care should be taken so that written agreements are clear and do not limit appropriate knowledge sharing.

Appendices to the guidance are available in the online version of the guidance document (6).

End of text from the EUPATI guidance on patient involvement with HTA.

______________________________________________

## Discussion

### New resources and research on patient involvement

The arguments for patient involvement in HTA are well rehearsed, and can be summarized as arising from the following considerations: (i) patient rights—as the ultimate beneficiaries, patients should be consulted on decisions about their healthcare; (ii) patient and community values—healthcare services should be aligned with the values of the patients they serve; (iii) patients contribute to evidence—patients' unique insight into living with their health condition, and the impact of treatments and services, adds to the evidence base of the HTA process; (iv) improving HTA methods—patient input can help identify outcomes that matter to patients and ensure they are included in scientific advice discussions and reported in HTA reports (7).

The EUPATI guidance document on patient involvement in HTA was first released in 2016. Further context for patients wishing to better understand HTA is provided by online articles, a short video and a recorded webinar on the EUPATI website^4^^,^^5^^,^^6^^,^^7^^,^^8^. There remain major regional variations in the levels of patient involvement in HTA, and in the support patients receive for such involvement. Some HTA agencies have been developing their processes over many years and have a stated policy of involvement with dedicated staff to support patient involvement. Others have no mechanisms for patient involvement or use simple methods of public consultation without any specific support to enable patients to participate meaningfully.

The HTAi Patient and Citizen Involvement Interest Group (PCIG) is working toward providing a comprehensive and searchable international directory of publicly available materials that have been specifically designed for patients to help them participate in HTA processes. The aim is to support organizations that are interested in involving patients and/or citizens in their HTAs; as well as to directly support patients and citizens themselves who wish to become involved and learn about their role in HTA. Materials will include information about the role of HTA processes, how HTA is embedded in the health system; information written for patients and the public on how and why they should be involved; and training and education resources for patients and the public about HTA in specific regions. The working prototype is scheduled for release in mid-2018 and will be available on the PCIG webpage^9^.

For academics and HTA professionals requiring in depth information, a new text book was published in 2017 covering patient participation and research into ways of taking account of patients' expertise and preferences (8). The book addresses the rationale for patient involvement, a guide to consistent terminology, discussion of approaches to participation and detailed descriptions of applicable research methods. A set of international case studies is also presented. The HTAi PCIG plans to use the structure and content of the book to develop a series of workshops and webinars to particularly assist regions just starting the journey of patient involvement.

In a useful complement to these initiatives, a “framework for action” for public and patient involvement (PPI) in HTA has been developed for a Canadian HTA organization and may be applicable to other similar health systems (9). The framework draws on international practice and published research and a consultation with a variety of stakeholders. It includes four “actionable elements”: (i) guiding principles and goals of PPI in HTA; (ii) establishment of a common language to support PPI; (iii) a flexible array of approaches; (iv) ongoing evaluation of PPI to drive improvement.

Evaluation of patient involvement in HTA, and of the impact it has, will be an important driver of improvements in the process (10). Recognizing the scarcity of evidence in this area, the HTAi PCIG recently surveyed HTA organizations internationally (11). They found that the number of organizations carrying out impact evaluation is still small (although difficult to accurately quantify due to a low response rate), that approaches to evaluation vary widely, but that the results of the evaluations in some cases lead to specific changes in patient involvement processes and enhanced awareness within the HTA organizations of patient involvement initiatives. The survey respondents shared insights into the challenges and facilitators for evaluation, including identifying some facilitators that have been recognized in the broader literature on evaluation, such as the need for explicit, measurable objectives and the inclusion of a range of stakeholders on evaluation teams.

Expanding on this work, Gagnon et al. provide an overview of current published practice in evaluation of patient involvement in HTA, and a discussion of the challenges identified in the wider literature. They recommend a framework for evaluation that considers the community, organizational, decision-making and political contexts (10).

Feedback directly to patients on how their submissions inform specific HTAs allows patients to make their own evaluation of their impact (and promotes transparency), and has been specifically called for by patient groups and others (3, 10, 12–16).

### Barriers to patient involvement in HTA

Some HTA organizations now have a wide range of involvement processes, allowing for patient input to design and improvement of HTA processes as well as submissions to individual HTAs. Some HTA organizations only allow public consultation in which patient organizations can participate. Others do not encourage any patient involvement. This often occurs because HTA is seen as a scientific process and patient input is considered anecdotal or biased. When patient involvement is encouraged there can still be barriers including lack of financial compensation, poor training and support, and low general awareness. The EUPATI HTA guidance document includes suggestions to address these potential pitfalls.

When patients have the opportunity to contribute to HTA activities they commit significant amounts of time and effort, and yet, as the guidance document points out, many do so in a voluntary capacity even when acting as representatives of patient organizations. Fair compensation for time, and reimbursement of expenses, is an essential part of facilitating patient involvement.

Similarly, patients are likely to need training and support that is carefully designed with their needs in mind, to build understanding, confidence and effectiveness. Support, for example a point of contact at HTA organizations and the facilitation of peer group networks, is as important as training in technical aspects of HTA.

Calls for proper compensation, and effective training and support, have been made by many stakeholders (1, 3, 12–16). Opportunities for involvement also need to be properly communicated by HTA organizations to patients. Patient organizations are encouraged to be proactive in approaching HTA organizations to begin discussions about involvement, but the onus is also on HTA organizations to be creative and reach out to new patient populations to ensure willing participants are not excluded.

## Actionable recommendations and conclusion

Despite calls for meaningful involvement of patients in HTA, the extent of patient involvement, and levels of support for patients, continue to vary hugely across Europe. Barriers to involvement, such as a lack of financial support, poor training and low awareness of existing involvement opportunities, remain. The EUPATI HTA guidance document, developed using existing literature and extensive internal and external consultation, remains an important source of actionable recommendations centered on suggested working practices (for HTA organizations) and patient involvement activities (for individual HTA processes). These are set out within the guidance text itself, presented in this paper. The suggested working practices for HTA organizations are categorized as outreach and education activities; how to achieve wider involvement; and resource provision. The patient involvement activities for individual HTA processes contribute to all stages of an HTA: identifying and prioritizing topics; scoping specific HTAs; assessing medicines; and reviewing and disseminating decisions. The guidance does not represent a call for a change to the existing levels of autonomy of HTA organizations but seeks to address the gap in European-wide guidelines on effective involvement of patients in HTA, providing a tool to promote involvement and overcome potential barriers across member states. It is complemented by a growing body of resources for patients, academics and HTA organizations, and by growing calls for HTA organizations to ensure that willing participants are effectively included.

## Author contributions

AH drafted the article and the guidance document with significant input from KF. VT, DH, KW, IK, MM, and WS reviewed and revised the article and guidance document. All authors were contributors to the IMI project: European Patients' Academy on Therapeutic Innovation (EUPATI).

### Conflict of interest statement

The authors declare that the research was conducted in the absence of any commercial or financial relationships that could be construed as a potential conflict of interest.

## Footnotes

^1^https://www.eupati.eu/guidance-patient-involvement/ Last accessed 24 May 2018.

^2^The term ‘patient' here refers to several definitions, including patient representatives: see “Defining “patient””

^3^In some European countries, carers are referred to as ‘caregivers'.

^4^https://www.eupati.eu/health-technology-assessment/fundamentals-of-health-technology-assessment-process/ Last accessed 5/12/17.

^5^https://www.eupati.eu/health-technology-assessment/the-role-of-health-technology-assessment-in-health-systems/ Last accessed 5/12/17.

^6^https://www.eupati.eu/health-technology-assessment/responsible-health-system-decision-making-and-hta/ Last accessed 5/12/17.

^7^https://www.eupati.eu/health-technology-assessment/patient-organisation-involvement-in-hta-processes/ Last accessed 5/12/17.

^8^https://www.eupati.eu/webinar/webinar-strengthening-patient-involvement-health-technology-assessment-hta/ Last accessed 5/12/17.

^9^HTAi Patient and Citizen Involvement Group webpage www.htai.org/interest-groups/patient-and-citizen-involvement.html Last accessed 16/11/17.
